# Tobacco-free campuses – a pipe dream? A survey of current smoking cessation practice in mental health units in Ireland

**DOI:** 10.1192/bjb.2023.50

**Published:** 2024-06

**Authors:** Colm Harrington, Elaine Walsh

**Affiliations:** 1 National University of Ireland, Galway, Ireland; 2 University Hospital Galway, Galway, Ireland; 3 Mayo University Hospital, Castlebar, Ireland

**Keywords:** Tobacco-free hospital campus, e-cigarette, smoking, addiction, vaping

## Abstract

**Aims and method:**

Smoking and vaping are prohibited on Ireland's Health Service Executive (HSE) campuses. The HSE states that there is no evidence to suggest vaping is less damaging than cigarettes. Recent meta-analyses have shown that e-cigarettes are in fact less dangerous and can help smokers quit. Our study analyses the current smoking policies in place in mental health ‘approved centres’ in Ireland, what is being done to help smokers quit while in-patients and the level of support among staff for the introduction of e-cigarettes as a harm reduction tool. Clinical nurse managers from each mental health approved centre were surveyed to assess adherence to smoking policies.

**Results:**

Only 5% of surveyed units enforce the HSE's Tobacco Free Campus Policy; 55% of units supported the idea of using e-cigarettes to help patients quit cigarettes.

**Clinical implications:**

Ireland's hospital campuses are not tobacco free. Changes need to be made to our smoking policies and their enforcement.

Smoking is the leading preventable cause of death and disease in the world and accounts for 4500 deaths and 44 000 hospital admissions annually in Ireland.^1^ Ireland's Health Service Executive (HSE) spends approximately €280 million a year treating tobacco-related diseases.^2^

In 2021, smoking prevalence in Ireland was 16.1%.^3^ This is down from 23% in 2016.^4^ Significant reductions in current smoking are required to meet the Tobacco Free Ireland 2025 target of <5% smoking prevalence.^5^

As per the HSE's Tobacco Free Campus Policy, smoking by employees, patients, visitors and any other parties has been prohibited on all HSE campuses since 31 December 2015.^6^ Compliance with this policy is poor. A recent audit showed that up to 11% of people observed on hospital grounds had a cigarette in their hand.^7^

According to the HSE report *Smoking Cessation and Mental Health*,^8^ smoking rates among adults with a common mental disorder such as depression or anxiety are almost twice as high compared with adults who are mentally well and three times higher for those with schizophrenia or bipolar disorder. People with substance use disorders, with or without a comorbid mental health problem, have the highest rates of smoking. In every area of mental health, even child and adolescent mental health services, perinatal psychiatry and older adult care, smoking rates are disproportionately high. People with a mental illness tend to smoke more heavily and are more dependent on nicotine than those without a mental illness. They are just as likely to want to stop smoking but often lack confidence in their ability to quit and historically have not routinely been offered specialist support to quit.^9^

People with mental health conditions die on average 10–20 years earlier than the general population, and smoking is the single largest factor accounting for this difference.^10^

Implementing strategies to reduce the high prevalence of smoking in people generally, and more specifically in smokers with mental disorders, will not only have a positive impact on quality of life, but also has the potential to realise cost savings for the HSE.^9^

Besides improvements to physical health, in common with the general population, people with mental illnesses who stop smoking often require lower doses of medication.^11^ This helps to minimise some of the negative impacts of these medications.

Furthermore, there is comprehensive evidence that smoking cessation is associated with reduced depression, anxiety and stress, and improved positive mood as large for those with mental health problems as those without, and the effect is equal or greater than those of antidepressant treatment for mood and anxiety disorders.^12^

The issue of the high burden of smoking and poor provision of smoking cessation support to people with mental illnesses has been identified internationally.^13^ Attitudes, expectations and culture in relation to smoking can be a particular challenge in mental health services.^14^ In Ireland, the mental health sector was exempted from smoke-free workplace legislation,^15^ and this challenge has also been associated with poor delivery of smoking cessation support to patients within secondary mental health services compared with other service settings.^16^

It has been demonstrated that, despite higher smoking prevalence compared with other patients, motivation to quit, acceptability of cessation advice and quit rates similar to those for other patients, provision of clinical care for tobacco use among patients in secondary mental health services was approximately one-third that seen in Irish general in-patient samples.^17^

Tobacco Free Ireland identifies persons with mental health problems as a priority group, as does the HSE Tobacco Free Ireland Programme Plan 2018–2021.^18^ The Department of Health's mental health policy also identifies the need to better respond to the particular physical needs of people with mental health problems.^19^

Table 1 shows data on smoking cessation and e-cigarette usage in Ireland from the Healthy Ireland Surveys for 2019 and 2021. An important finding is that 44% of smokers had attempted to quit in 2021,^3^ although only 25% of attempts to quit reported in 2019 were successful.^4^

**Table 1:** Overview of smoking cessation and e-cigarette usage in Ireland, 2019^4^ and 2021^3^

Ex-smokers	28%
Smokers who attempted to quit in past year	44%
Attempts to quit that are successful	25%
Successful and employed will power alone	52%
Current smokers using e-cigarettes	10%
Ex-smokers using e-cigarettes	13%
Used e-cigarettes to quit smoking	38%
Use e-cigarettes and never smoked	1%

Source: Healthy Ireland Survey reports, 2019 and 2021.^3,4^

For nicotine replacement therapy the HSE recommends gum, lozenges, patches, inhalers and mouth sprays.^20^ The HSE's e-cigarette policy states ‘There are no properly conducted scientific studies to prove that e-cigarettes are an effective aid for sustained smoking cessation’.^20^ It goes on to report that ‘there is a legitimate concern that, because e-cigarettes resemble ordinary cigarettes, their use may promote smoking. Accordingly, they should be prohibited in the same way that tobacco products are. Patients and members of the public should be advised that we cannot say that they are safe and so they may not be used within the bounds of any health service campus’.^20^ However, in the UK, Public Health England have stated that there is stronger evidence in this year's report that nicotine vaping products are effective for smoking cessation and reduction.^21^

It has been found that e-cigarette users have greater concentrations of biomarkers of nicotine, tobacco-specific nitrosamines, volatile organic compounds and metals compared with people who have never used tobacco. However, these concentrations were significantly lower than those observed in cigarette smokers.^22^

In 2014, an international expert panel convened by the Independent Scientific Committee on Drugs published a report stating ‘Cigarettes are the nicotine product causing by far the most harm to users and others in the world today. Attempts to switch to non-combusted sources of nicotine should be encouraged as the harms from these products are much lower’.^23^

In 2019, a *New England Journal of Medicine* editorial stated that ‘a consensus has emerged that e-cigarettes are safer than traditional combustible cigarettes’.^24^ It has been argued that ‘harm reduction’ approaches should be used, encouraging smokers to switch to e-cigarettes. England and New Zealand are taking a harm reduction approach, encouraging or supporting smokers to switch to vaping.

Public Health England's seventh independent report on vaping in England, carried out by researchers at King's College London, found that nicotine vaping products were the most popular aid (27.2%) used by smokers trying to quit in England in 2020.^21^ It is estimated that in 2017, more than 50 000 smokers stopped smoking with the aid of a vaping product who would otherwise have carried on smoking. In 2020, 38% of surveyed smokers believed that vaping is as harmful as smoking – 15% believed that vaping is more harmful. Using a vaping product as part of a quit attempt in local smoking cessation services had some of the highest quit success rates – between 59.7% and 74% in 2019–2020.^21^ The UK Action on Smoking and Health Report stated that e-cigarette use among 11- to 18-year-olds has to date remained low, but on the downside their potential as an adult quitting aid has not been fully realised.^10^

There is strong evidence from multiple randomised controlled trials that nicotine replacement therapy, bupropion, varenicline, face-to-face behavioural support, telephone support, interactive websites and written self-help materials can increase smoking cessation rates when used in a quit attempt.^25^ According to the 2021 Cochrane Review on e-cigarettes for smoking cessation, ‘There is moderate certainty evidence that e-cigarettes with nicotine increase quit rates compared to nicotine replacement therapy’.^26^ The review authors did not detect evidence of harm from nicotine e-cigarettes, but the longest follow-up was only 2 years.

## Aim and objectives

Despite HSE campuses being officially tobacco free, cigarette smoking is a common sight outside hospitals and especially mental health units. Our research aimed to analyse the current smoking policies in place in the mental health approved centres in Ireland. The objectives were to (a) measure adherence to the official policy and (b) identify what is being done to help smokers quit while in-patients. We also wanted to gauge the level of support among staff for (c) a tobacco-free campus and (d) introduction of e-cigarettes as a harm reduction tool.

## Method

It was planned to contact the clinical nurse managers in charge of each of mental health approved centre in Ireland. A list of 57 approved centres was identified on the Mental Health Commission website. The phone numbers for each of these units were found on the HSE website. A brief telephone survey was used to assess adherence to smoking policies and measures employed to reduce cigarette smoking by in-patients. The questionnaire was developed by the researchers and had 13 questions (Appendix). It took approximately 15 min to complete. One investigator performed the data collection and data analysis, thereby increasing internal validity. Where participants wanted to offer additional information, we made note of this. The nominal data were collated and analysed. The survey was performed in 2022.

### Ethical approval

The author asserts that all procedures contributing to this work comply with the ethical standards of the relevant national and institutional committee on human experimentation with the Helsinki Declaration of 1975, as revised in 2008. The author asserts that ethical approval for publication of this research has been provided by their local Ethics Committee (Mayo University Hospital).

## Results

Of the 57 approved centres, 40 (70.2%) were included in the study where there was a clinical nurse manager available to respond to the survey questionnaire.

Results are outlined in Table 2. Eight units (20%, *n* = 40) reported that they were not tobacco-free sites; the remaining 32 units (80%) reported that they were tobacco free. Only two units (5%) reported that they were strictly enforcing the tobacco-free site policy. Thirty-eight units (95%) allowed patients to smoke in designated areas on the tobacco-free campus. Examples of these designated areas were enclosed gardens attached to the unit.

**Table 2:** Outline of results of survey of mental health approved centres in Ireland

Response to survey	70%
Tobacco free policy enforced	5%
E-cigarette ban	100%
Smoking cessation officer	65%
Nicotine replacement offered	100%
Nicotine replacement usually rejected	70%
Staff threatened by patients when restricting smoking	55%
Risk incidences from cigarettes or lighters	62.5%
Risk incidences from e-cigarette use	2.5%
Support for e-cigarette use as harm reduction tool	55%

All units reported having a ban on e-cigarette use, but again e-cigarettes were allowed in the 38 units that had designated smoking areas.

Twenty-six units (65%) had access to a smoking cessation officer. Fourteen units (35%) did not. All 40 units reported offering nicotine replacement therapy routinely when admitting patients, but 28 units (70%) stated that the replacement therapy offered was rarely taken up by the patient. Nine units (22.5%) stated that it was usually taken up and two units reported an uptake rate of around 25%. One unit stated that it was usually only taken up by patients who were in isolation and could not access the smoking area.

Nicotine patches were the most common form of nicotine replacement, with 39 of the 40 units (97.5%) offering them. Twenty-eight units (70%) offered nicotine inhalers. Twenty-five units (62.5%) had nicotine gum. Nine units (22.5%) used nicotine spray.

Twenty-one units (52.5%) reported that they commonly had patients complaining about not being allowed to smoke. This occurred when the designated smoking areas were closed at night or patients were in isolation or seclusion. Of the 19 units (47.5%) that did not have patients complaining about not being allowed to smoke, the reason given was that there were no restrictions on smoking. Two units reported frequently receiving complaints from non-smokers about the high levels of second hand smoke, which made it difficult for them to enjoy the enclosed outdoor areas.

Twenty-two units (55%) reported having had nursing staff threatened by patients who had restrictions put on their smoking.

Twenty-five units (62.5%) had had risk incidences involving cigarettes or cigarette lighters (e.g. self-harm and intentional damage to property). Two units mentioned having to have furniture and wallpaper changed because of damage from smoke exposure.

Only one unit reported a risk incident involving e-cigarettes and that was having a fire alarm triggered by e-cigarette smoke. Another unit expressed concerns about the potential use of e-cigarettes as a weapon, as some models can be quite weighty.

Twenty-two units (55%) stated that they would support the use of e-cigarettes as a harm reduction tool to help patients quit smoking and maintain a strict tobacco-free campus. Fourteen units (35%) said they would not support this. Four units (10%) said they did not know whether there was enough evidence to support this. One unit had helped several patients quit smoking by procuring e-cigarettes for them with the patients’ money.

## Discussion

This novel study shows that Ireland's hospital campuses are not tobacco free despite the HSE declaring them so in 2016. Mental health units are supposed to be tobacco free but this is not enforced in most approved centres, despite psychiatric patients being particularly vulnerable to the deleterious physical and mental health effects of tobacco smoking.

Cigarette smoking also causes issues for non-smoker in-patients who share common spaces with smokers and have to deal with second hand smoke. They may also be harmed in risk incidences involving cigarettes and cigarette lighters, which this study shows occur commonly. Smoking continues to cause problems for staff too, as most had experienced being threatened by patients who were not allowed to smoke.

The uptake of nicotine replacement therapies was very poor. This is likely to be because the patients could continue smoking cigarettes.

One of the units that had a strict no smoking policy stated that their patients accepted this well and they had a high uptake of nicotine replacement therapies.

To reach the Tobacco Free Ireland 2025 target of <5% smoking prevalence and to look after the physical and mental health needs of our patients, changes need to be made to our smoking policies and how they are enforced. Making smoking cessation a routine part of psychiatric care plans would allow lower doses of psychiatric medications to be used, thus decreasing risk of side-effects.

Tobacco-free campuses must be properly enforced, with the removal of designated smoking areas. This would provide patients the impetus to take up nicotine replacement therapy to help them quit smoking. It is not necessary for patients to experience nicotine withdrawal when quitting smoking. Nicotine replacement therapies must be attractive and provide adequate relief from nicotine withdrawal.

E-cigarettes are being shown to be the most popular method for smoking cessation by smokers in the community. With the most up-to-date evidence showing e-cigarette use to be much less harmful than cigarette use, perhaps now is the time to change smoking policies to reflect this and give our patients the best chance to quit cigarettes.

Further research to explore the barriers to enforcing tobacco-free campuses would be helpful.

### Key points

- This is the first study of its kind in Ireland.
- ‘Tobacco-free campuses’ are poorly enforced.
- Reduction in smoking has potentially significant benefits for mental health patients, with a consequential reduction in the use of medications, side-effects and medication spending, and an improved quality of life for patients.
- Consideration should be given to the use of e-cigarettes as part of a smoking cessation programme.

## Data Availability

Data are available on reasonable request from the corresponding author, C.H.

## About the authors

**Colm Harrington**, MB BCh, BAO, MCPsychI, MRCPsych, DTMPH, is a psychiatry registrar in the Adult Acute Mental Health Unit, University Hospital Galway, Galway, Ireland. **Elaine Walsh**, MB BCh, MRCPsych, is a consultant in psychiatry of later life at Mayo University Hospital, Castlebar, Ireland.

## Appendix

### Smoking policy questionnaire

1. Is your hospital a tobacco-free campus?
2. Are there designated places on campus where patients are still allowed to smoke?
3. Are people allowed to use e-cigarettes on your campus?
4. Is nicotine replacement therapy routinely offered?
5. Is nicotine replacement therapy routinely taken up by patients?
6. What nicotine replacement do you offer (inhalers, gum, losenges, patches)?
7. Are e-cigarettes encouraged as a smoking cessation strategy?
8. Do patients complain about not being allowed to smoke?
9. Have staff been threatened by patients who are not allowed to smoke?
10. Would you support the use of e-cigarettes on campus as a harm reduction strategy?
11. Have there ever been risk management issues caused by e-cigarettes on campus?
12. Have there ever been risk management issues caused by cigarettes or lighters on campus?
13. Do you have a smoking cessation specialist?
